# Facing the SARS-CoV-2 Outbreak: What Should Obstetricians and Gynecologists Do?

**DOI:** 10.1017/dmp.2020.263

**Published:** 2020-07-27

**Authors:** Hongyuan Zhang, Yuanjing Hu, Yingjun Zhu, Xu Chen

**Affiliations:** Tianjin Key Laboratory of Human Development and Reproductive Regulation, Department of Gynecology, Tianjin Central Gynecology and Obstetrics Hospital, Nankai University, Tianjin, China

**Keywords:** COVID-19, experience, gynecologist, obstetrician, SARS-CoV-2

## Abstract

An outbreak of coronavirus disease 2019 (COVID-19) caused by the severe acute respiratory syndrome coronavirus 2 (SARS-CoV-2) has spread worldwide. In this major outbreak, women are a special group, especially pregnant patients. Many problems faced by clinicians are still unclear and need to be solved. As the largest obstetrics and gynecology hospital in North China, here we summarize the diagnosis and treatment process and key points of obstetrics and gynecology patients in our hospital during the period of the COVID-19 pandemic, hoping to provide available information to inform care of obstetrics and gynecology patients.

Coronavirus Disease 2019 (COVID-19) is an emerging disease with a rapid increase in cases and deaths since its first identification in Wuhan, China, in December 2019. By June 3, 2020, it has resulted in more than 6 360 000 infections and over 380 000 deaths worldwide. The World Health Organization declared a public health emergency of international concern (PHEIC) on January 30, 2020, and characterized the COVID-19 situation as a pandemic on March 11, 2020, in response to the reports of human-to-human transmission and rapid growth of the outbreak.^1^

There has been a rapid increase in knowledge of the genetic, virologic, epidemiologic, and clinical aspects of this emerging agent. In this pandemic, women are a special group, especially pregnant patients. Many problems faced by clinicians are still unclear and need to be solved. Not only is this a significant public health issue, but also it represents an obstetrical and gynecological management issue in determining the care received by female patients. Here, we summarize some experiences and deficiencies of our hospital during the period of COVID-19 pandemic, hoping to provide available information to inform care of obstetrics and gynecology patients.

## CLINICAL FEATURES OF PREGNANT WOMEN WITH COVID-19

According to the clinical data currently reported, the clinical characteristics of patients with COVID-19 infection during pregnancy were similar to those of non-pregnant adults with COVID-19 infection.^2,3^ There is a theoretical risk of vertical transmission, as the angiotensin-converting enzyme 2 (ACE2) receptor is widely expressed in the placenta, but there is currently no evidence to suggest that the development of COVID-19 pneumonia in the third trimester of pregnancy could lead to the occurrence of severe adverse outcomes in neonates and fetal infection that might be caused by intrauterine vertical transmission. It is notable, however, that the overwhelming majority of the studies were small sample sizes and the patients were recruited in the third trimester, so it is not likely to ascertain the possibility of intrauterine vertical transmission during the first or second trimester.

Theoretically, pregnant women are particularly susceptible to respiratory pathogens and severe pneumonia, because they are at an immunosuppressive state. In addition, functional residual capacity, end expiratory volumes, and residual volumes decrease steadily from early pregnancy due to diaphragm splinting by the gravid uterus, resulting in reduced total lung capacity at term and an inability to clear pulmonary secretions effectively; physiological adaptive changes during pregnancy (eg, diaphragm elevation, increased oxygen consumption, and edema of the respiratory tract mucosa) can render them intolerant to hypoxia.^4^ In the context of the pulmonary changes described previously, it would more readily predispose to hypoxemic respiratory failure in pregnancy. Previous data on severe acute respiratory syndrome (SARS) and Middle East respiratory syndrome (MERS) suggest that clinical findings during pregnancy can range from no symptoms to severe disease and death. But, until now, there are no data to inform whether pregnancy increases susceptibility to COVID-19.

## OUR MAIN MANAGEMENT IN PERINATAL PERIOD IN COVID-19

- Universal COVID-19 testing was initiated for all women admitted to the labor unit for delivery, as well as for women admitted for antepartum indications and postpartum complications, with the purpose of improving identification, preventing exposure, and guiding clinical management.
- Comprehensive laboratory tests include a complete blood count, liver function tests, serum creatinine level, blood type, lactate dehydrogenase, inflammatory markers (C-reactive protein, ferritin), coagulation tests (D-dimer, fibrin degradation products, prothrombin time, activated partial thromboplastin time), and procalcitonin.
- For patients with negative reverse transcription polymerase chain reaction (RT-PCR) test and highly suspected COVID-19 infection, with informed consent obtained, repeat RT-PCR test and carry out lung CT examination at the same time. Because pregnant patients could be particularly sensitive to exposure dose, the patient’s lower abdomen and pelvis were covered with a lead blanket before CT examinations,^5^ and the low-dose imaging mode was used.^6^
- Ensure rapid triage of pregnant patients with respiratory symptoms. For clinically stable women diagnosed with infection, an outpatient monitoring is required, and if relevant symptoms or findings appear, nursing upgrading approaches (including hospitalization) are required.^7^
- For suspected cases of COVID-19, the patients should be isolated to a negative pressure room or isolation ward as soon as possible and wait for the result of the nucleic acid test.
- A dedicated negative pressure operating room was established for pregnant women who must deliver with confirmed COVID-19 infection, and the newborns should be isolated in different rooms and be screened very carefully.
- Streamline medical care providers into single groups, each comprising the attending, resident, intern, and nursing or midwifery staff. The individual teams function independently. If a team member is exposed to or infected with COVID-19, their team will be quarantined for at least 2 weeks.
- Oxygen inhalation to keep oxygen saturation above 95%. Oxygen may be given via high-flow or non-rebreather mask, according to the patient’s clinical condition.
- Monitor the fetus with at least 1 ultrasound each week and once a day of electronic fetal heart rate monitoring to evaluate the fetal growth and intrauterine hypoxia, as the changes in fetal heart rate pattern may be an early indicator of maternal respiratory deterioration.^8^
- Corticosteroids are not used routinely because they were not shown to be beneficial in MERS and could lead to delayed MERS-CoV clearance.^9^ Administration of betamethasone should be considered to promote fetal lung maturity when preterm delivery is anticipated.
- Empiric antimicrobial therapy to prevent secondary bacterial infections and antibiotics should be tailored to drug sensitivity results.
- Use intravenous fluids conservatively to avoid fluid overload unless cardiovascular instability is present. If septic shock is suspected, institute prompt, targeted management.
- The mode of delivery is directed by obstetric factors and clinical urgency. Vaginal delivery is not contraindicated in patients with COVID-19, as the studies showed that neither type of delivery affected newborns and all of the studied newborns were negative for COVID-19 infection.^10,11^ When emergent delivery is required in a critically ill parturient, a cesarean section is most appropriate.
- Suck the nasopharynx before the first breath of the baby, as we should pay special attention to prevent infections in newborn babies born to mothers with COVID-19 pneumonia.
- No delayed cord clamping of the umbilical cord, so as to clean the mother’s blood and amniotic fluid as soon as possible after the baby’s birth.
- Infected or suspected mothers should avoid breastfeeding until she recovers completely or has been confirmed not to have COVID-19 as the infected mother can transmit the COVID-19 virus through respiratory droplets during breastfeeding.

## OUR MAIN MANAGEMENT OF GYNECOLOGICAL CANCER PATIENTS

Gynecological cancer patients are more susceptible to severe acute respiratory syndrome coronavirus 2 (SARS-CoV-2) infection due to the systemic immunosuppressive state caused by the malignancy itself, or surgery/radiotherapy and chemotherapy. Standardized management should be implemented for gynecological cancer patients to achieve scientific and accurate protection.

A thorough physical examination of all inpatients, including assessment for fever, and a pulmonary examination are critical. Given the spectrum of tumors encompassed by gynecological oncology, treatment of aggressive or malignancies needs to be individualized according to the tumor, patient, and health care resource considerations. In case of life-threatening conditions, such as rupture or bleeding of a gynecological tumor, emergency operation and treatment should be carried out on the premise of adequate protection. The epidemic investigation should be further improved when the condition is stable. Patients with an advanced tumor or rapid progress of disease should be treated as early as possible and diagnosed clearly, and those who have the opportunity of operation should pursue it as soon as feasible. For patients with precancerous lesions, such as cervical intraepithelial lesions, we recommended deferring treatment for 3 months.

In the patients who tested positive for COVID-19 infection or suspected patients, the choice of surgical approach should be cautious. Because of the pneumoperitoneum in laparoscopic surgery, viral particles in the surgical plume could potentially escape into the operating theater from leakage around an imperfect trocar seal and during rapid venting through the trocars at the time of changing instruments, removing specimens, or desufflation at the conclusion of the operation.^12,13^ We have taken some measures to reduce the risk of infection in laparoscopic surgery, including operating at a lower pressure (10–12 mmHg) pneumoperitoneum, if possible; avoiding leakage or blood spray from trocar sites; avoiding rapid venting from the trocars; using a laparoscopic suction with a filter system to avoid rapid venting before changing instruments; removing specimens; and removing trocars.

For patients with severe myelosuppression, fever, or hemorrhage, or the elderly and other high-risk groups receiving chemotherapy for malignant tumors, granulocyte colony stimulating factor (G-CSF) and granulocyte macrophage colony stimulating factor (GM-CSF) can be used prophylactically after chemotherapy to avoid leucopenia and improve immunity. Fever after operation or chemotherapy should be differentiated from SARS-CoV-2 infection. A CT examination should be performed again, if necessary, and an inquiry made to the infection department for consultation. If SARS-CoV-2 infection is not excluded, it should be detected again and with isolation of the infected patients as soon as possible.

## CONCLUSION

With the global spread of COVID-19, there will be additional information available on the effects of COVID-19 on obstetrical and gynecological patients, especially on pregnant women. This narrative represents an integrated framework to provide an appropriate level of care for these patients and hospital staff during the COVID-19 pandemic. We need to further strengthen research to provide an evidence-based foundation for the medical management of obstetrical and gynecological patients with COVID-19.
